# Unmet needs of people with epilepsy: A qualitative study exploring their journey from presentation to long-term management across five European countries

**DOI:** 10.3389/fneur.2023.1130817

**Published:** 2023-04-14

**Authors:** Ella Graham-Rowe, Caroline Brigitte Katzer, Sumira Riaz, Amanda Attwood, Liz Bates, Ricardo Sainz-Fuertes, Becky Swan

**Affiliations:** ^1^OPEN Health Communications LLP, Marlow, Buckinghamshire, United Kingdom; ^2^Eisai Europe Ltd, Hatfield, United Kingdom

**Keywords:** epilepsy, patient journey, emotions, qualitative, market research, unmet needs

## Abstract

**Introduction:**

Epilepsy is a neurological disease that can negatively impact a person’s physical, psychological, social, and emotional well-being. The aim of this study was to provide insights into the experiences of people with epilepsy on polytherapy (i.e., people on a combination of two or more anti-seizure medications [ASMs]), with an emphasis on their emotional journey.

**Methods:**

Market research was conducted with 40 people with epilepsy from France, Germany, Italy, Spain, and the United Kingdom. Semi-structured interviews were analyzed using both a content and framework analysis approach. A content analysis of participants’ expressed emotions was used to illustrate the changes of emotions experienced by people with epilepsy from presentation through to monitoring and follow-up stages.

**Results:**

In each stage of the journey, themes and subthemes were identified under the overarching headings: Stage 1: Presentation – Life is turned upside down; Stage 2: Diagnosis – Period of learning; Stage 3: Treatment – Aspirations and experimentation; and Stage 4: Monitoring and follow-up – Feeling “out on a limb”. The research identified key unmet needs and opportunities for people with epilepsy to improve their subjective experiences at different stages of their disease journey, namely: (1) establish and promote support networks from presentation through to monitoring and follow-up stages; (2) accelerate pathway to diagnosis; (3) provide opportunities to discuss the diagnosis with patients; (4) clarify treatment-change guidelines for patients; and (5) develop a shared treatment decision-making/empowerment tool.

**Discussion:**

The research findings and recommendations have the potential to drive change at an individual level, as well as at a healthcare level.

## 1. Introduction

Epilepsy is one of the most common and debilitating neurological conditions, and it has been estimated to affect between 50 and 75 million people globally (1, 2). The daily burden for people with epilepsy who experience epileptic seizures and accompanying symptoms remains high, despite advances in understanding pathophysiological disease mechanisms and treatment options (3). As well as the physical implications of epilepsy, such as disability, mortality, and comorbidities, the disease can also impact the psychological well-being and social aspects of peoples’ lives, including work, personal relationships, and quality of life (QoL) (4). Previous research has shown that emotions are closely related to the health and well-being of people with chronic disease (5), yet emotions are often overlooked in the literature reporting the subjective experience of people with epilepsy (6).

There are several qualitative and mixed-method research studies that have investigated patients’ subjective experiences (thoughts and feelings) of being diagnosed and living with epilepsy (7–17). The pathway to adjustment following a first seizure can trigger psychological concerns/issues, often stemming from the person’s perceived loss of control (16). Loss of control is an area of impact of living with epilepsy frequently reported in the qualitative literature. It impacted not only adults, but also children and adolescents, and was reported to be connected to fear of seizure recurrence, loss of control over their own bodies, as well as disruption to personal goals and plans (17). How quickly a person was able to adapt and re-establish perceived control following diagnosis could depend on their gender and clinical factors (e.g., presence of premorbid psychological disorder) (16). For example, people who were evaluated to have experienced a pervasive loss of control were thought to have a higher awareness of their own vulnerability and mortality, and have a higher fear of seizure recurrence and mood disturbances. They consequently needed to use more extensive strategies and external support to help them return to baseline levels of perceived control compared with those experiencing a limited loss of control following diagnosis (16).

Yennadiou and Wolverson (17), investigated how people of advanced age (more than 65 years) make sense of their epilepsy, and found that they appraised epilepsy as a powerful negative external force that is both threatening and unpredictable, yet perceived as separate from themselves. They also experienced loss of control, loss of independence, and difficulties dealing with stigma (17). Within this literature, social stigma is a common theme (10, 11, 17). Social stigma and persistent public misperceptions about epilepsy (e.g., the public perception that people with epilepsy are “possessed”), could have a disruptive effect on the person’s self-identity (10). Stigma could also impact a person’s self-esteem and social standing (10).

The findings illustrate the rich insights that qualitative accounts can provide, and how such techniques are invaluable when little is known about a particular issue or topic.

The current study was carried out as part of a wider program of research that aimed to provide a comprehensive, qualitative overview of the journey of living with epilepsy. The first step was a qualitative netnographic study of conversations posted on public social media sites relating to living with epilepsy (18). The analysis of these conversations identified key themes, namely: a lack of disease awareness among the public; the negative psychological and physical impact of seizures; the importance of ensuring appropriate sleep duration and quality; a tendency to understand disease burden through time (e.g., people with epilepsy were more likely to use the term “days” when describing negative experiences and “years” when describing positive experiences, especially when they were referring to treatment); the challenge of finding the right treatment and managing side effects; and the challenge of dealing with depression and anxiety (18). This was followed by a review of the published literature, reporting the emotional and clinical pathway of people with epilepsy and their carers (unpublished). This review identified themes relating to the impact of the relationship with a healthcare professional; stigma and how it can impact a person’s identity and self-esteem; the negative impact of epilepsy on everyday life/QoL; the experience and impact of seizures, symptoms, and treatment; a loss of independence; and mental health issues. Both pieces of research have highlighted that people with epilepsy have difficulties when first-line monotherapy treatment is not successful. This finding was supported by a recent published ethnographic study that explored the experiences of people who had either been diagnosed with drug-resistant epilepsy or had tried two or more anti-seizure medications (ASMs) without perceived success (19). The authors identified patient–provider gaps in both epilepsy and drug-resistant epilepsy treatment and management, and discovered that there was a negative impact of untimely disease management leading up to and after receiving a drug-resistant epilepsy diagnosis.

In this study, we recruited people for whom first-line monotherapy alone was not successful, and who were currently on a combination of two or more ASMs. Selecting this cohort of people with epilepsy, allowed us to not only identify the psychosocial consequences of epilepsy as they progressed through their clinical journey, but also to deep dive into the potential issues that can arise at the treatment and monitoring stages when monotherapy is not successful. To the best of our knowledge, the current study is the first to examine the experiential journey of people with epilepsy from the perspective of those on polytherapy, with a specific emphasis on their subjective experiences.

The aim of this study was to identify and raise awareness of the challenges and unmet needs faced by patients living with epilepsy on polytherapy in five European countries, and to identify opportunities to address those unmet needs.

The objectives were to:

- Understand the subjective experiences of patients living with epilepsy and how these experiences may change over the duration of the patient journey from presentation through to ASM treatment, and ASM treatment monitoring and follow-up stages
- Identify the impact these experiences have on patients’ everyday QoL (e.g., work life and relationships) and psychological well-being (e.g., emotions and mental health)
- Identify patients’ unmet needs at each stage of the patient journey and recommend opportunities to address those.

## 2. Materials and methods

### 2.1. Study design

Semi-structured interviews were conducted to explore the participants’ subjective experiences; for example, emotions (mental states brought on by neurophysiological changes, variously associated with thoughts, feelings, behavioral responses, and a degree of pleasure or displeasure) (20, 21), and feelings (a conscious experience created after the physical sensation or emotional experience) at each stage of the clinical journey (22, 23). This study was conducted as market research; therefore, ethical approval was not required. Codes of conduct/guidance for the (pharmaceutical) market research industry, including the British Healthcare Business Intelligence Association and the European Pharmaceutical Market Research Association were strictly followed. A team of market researchers and health psychologists, who are experts in strategic patient innovation and engagement, worked in conjunction with senior staff from a pharmaceutical company to design the study and analyze the qualitative research.

### 2.2. Participants and sampling procedures

A third-party recruitment company was used to recruit participants within the predefined criteria as follows:

1. Live in France, Germany, Italy, Spain, or the United Kingdom (UK)
2. Aged more than 18 years old
3. Diagnosed with epilepsy
4. Currently on an ASM combination adjunctive therapy for their epilepsy (i.e., at least on two ASMs, regardless of the disease duration and number of ASMs used previously)
5. Comfortable to talk about their personal experiences of being diagnosed with epilepsy and living with the condition – particularly around the emotions felt at different stages

In the context of this research area, we have defined polytherapy to mean when first-line monotherapy alone was not successful.

### 2.3. Interview procedure

Participants were invited to take part in a market research study about their experiences of being diagnosed with epilepsy and living with the condition, with a focus on their emotional experiences.

The interview topic guide was developed in English by the research team, and all United Kingdom interviews were moderated by the same team of researchers. Interview topic guides were then translated into French, German, Italian, or Spanish by native speakers through a third-party contracted recruitment company, and interviews in each of the four non-English speaking countries were carried out by local language moderators. The third-party were also responsible for recruiting participants from all five countries according to the pre-established inclusion criteria. Interviews were conducted *via* a web-assisted platform (Microsoft Teams) and lasted 60 min on average. All 40 interviews were recorded with permission and transcribed. Microsoft Excel was used to store, manage, and carry out the analysis of interview transcripts. Before the interview commenced, participants were read an introduction to the study, which provided details of (1) the study procedure; (2) confidentiality; (3) the right to withdraw or to refuse to answer any questions; and (4) the need for reporting adverse events. The interview topic guide is described in Table 1.

**Table 1:** Interview structure and examples of questions from the topic guide.

Section	Content overview/example questions
Section A: Set-up and introductions	Introduction to the study reporting adverse events
Section B: Warm-up and background	As an introduction, can you start by telling me a bit about yourself? Approximately when were you first diagnosed with epilepsy?
Section C: Diagnosis	In your own words, can you please tell me about your experiences before diagnosis? What questions and concerns did you have before you received your diagnosis? What prompted you to go to a healthcare professional?
Section D: Treatment	Tell me a little bit about when you first started add-on therapies (adjunctive therapy; i.e. two or more medications) Which healthcare professional spoke to you about changing treatments? To what extent did you look for additional information yourself about the chosen treatment?
Section E: Maintenance and monitoring	How often do you see healthcare professionals in relation to the ongoing management of your epilepsy? Do you have different consultations for different things (e.g., treatment, monitoring, ongoing symptoms management, reviews, *ad-hoc* appointments)? Which healthcare professional would you consider to be in charge of your condition?
Section F: Living with epilepsy	To begin with, how would you describe living with epilepsy? What emotional/psychological support has been offered to you (if any)? Thinking about living with epilepsy day to day, could you say how you currently feel emotionally?
Section G: Closing thoughts	Looking back over the time since you were first diagnosed with epilepsy, what aspects of life have been the most challenging for you? Why? What is your hope/wish for the future?

### 2.4. Data extraction and analysis

We followed a combined framework and content analysis approach. Framework analysis is a comparative form of thematic analysis using a structure of inductively and deductively derived themes, and is a popular approach primarily used in applied research (24). It is particularly suitable when there are more than one researcher analyzing the data, as it sets out a systematic method to data analysis, and can be adapted for use with both an inductive and deductive type of qualitative analysis (25). The framework approach used in the current study incorporated three core steps: (1) deductive analysis (coding data into the stages of a predefined epilepsy clinical journey map, which was initially based on clinical guidelines for each of the included countries); (2) content analysis (to identify emotions within the patient journey); and (3) inductive analysis (an in-depth thematic analysis of the participants’ accounts within each stage of the patient journey).

#### 2.4.1. Step 1: Identifying the clinical patient journey (deductive analysis)

The first step was the development of a map representing the distinct stages of a clinical journey in epilepsy, from presentation through to monitoring and follow-up stages. This was developed using insights derived from existing literature contained in clinical guidelines reporting the patient pathway within the five countries included in the research (26–30), as well as insights from the current research.

Quotes, extracted from the interview transcripts, were coded according to which stage of the clinical map they were evaluated to represent.

#### 2.4.2. Step 2: Identifying emotions within the patient journey (content analysis)

Content analysis of emotional expressions used by participants (positive or negative) was conducted. Similar emotions or emotional impact that were described or mentioned by the patients using different words were extracted from the interviews and grouped together, and an overarching emotional label was assigned by three health psychologists by consensus. For example, words/terms such as “nervous,” “anxious,” “tense,” “worried,” “scared,” or “afraid” would be grouped under the emotional label “fear,” or words/terms such as “understanding,” “reassured,” or “trusting” would be grouped under the label “reassured.” All overarching emotional labels (e.g., those that were reported by one or more participants) within each clinical stage (Step 1) were positioned on the Y axis of the map in descending order, starting with what was assigned by the research team (by consensus) to be the most positive (i.e., acceptance) down to the emotional label evaluated to be the least positive (i.e., anxious). The overarching emotional labels for each clinical stage were then visually mapped onto the correct coordinates of the graph/map (e.g., where the two points between the clinical stage [X axis] and the emotional label [Y axis] meet [the origin]).

#### 2.4.3. Step 3: Conducting an in-depth thematic analysis of patient experiences (inductive analysis)

Thematic analysis was used to explore participants’ experiences and to identify key emerging challenges and unmet needs (31, 32). Extracted data (i.e., any patient quotes relating to the impact of epilepsy on everyday life or insights into the patients’ subjective experience of living with epilepsy and/or their mental health) were examined and sorted within each prespecified stage of the clinical journey (e.g., presentation, diagnosis, and treatment), to synthesize and identify emerging content themes. Initial theme labels (describing broad content themes), and where relevant, sub-theme labels (nested within content themes) were then generated for each cluster of similar data to express these shared experiences.

## 3. Results

### 3.1. Participant characteristics

Forty participants were recruited in total, eight from each of the participating countries (France, Germany, Italy, Spain, and the United Kingdom). All participants were adults at the time of the interview, aged 18–65 years. Life stage of diagnosis varied, with some diagnosed in infancy/childhood and others in adulthood. Participants’ time of diagnosis spanned four decades from the 1980s up until the 2020s. Presentation also varied; participants reported experiencing several seizure types, including: absence, tonic–clonic, focal (aware and unaware), and myoclonic (Table 2). All participants were on polytherapy.

**Table 2:** Participant characteristics.

Screening questions	Responses	Number of participants
Type of seizure (most commonly experienced)	Focal seizures (aware)	3
	Focal seizures (unaware)	4
	Tonic–clonic seizures	11
	Tonic seizures	N/A
	Clonic seizures	N/A
	Absence seizures	7
	Myoclonic seizures	2
	Atonic seizures	N/A
	Other/or a mix of more than one of the above	13
Year diagnosed	1970–1979	2
	1980–1989	6
	1990–1999	8
	1990–1999	8
	2010–2019	12
	2020 onwards	4
Current age (range), years	18–25	3
	26–35	5
	36–45	13
	46–55	17
	56–65	2

N/A, not applicable.

### 3.2. Clinical patient journey map

**Figure 1:**
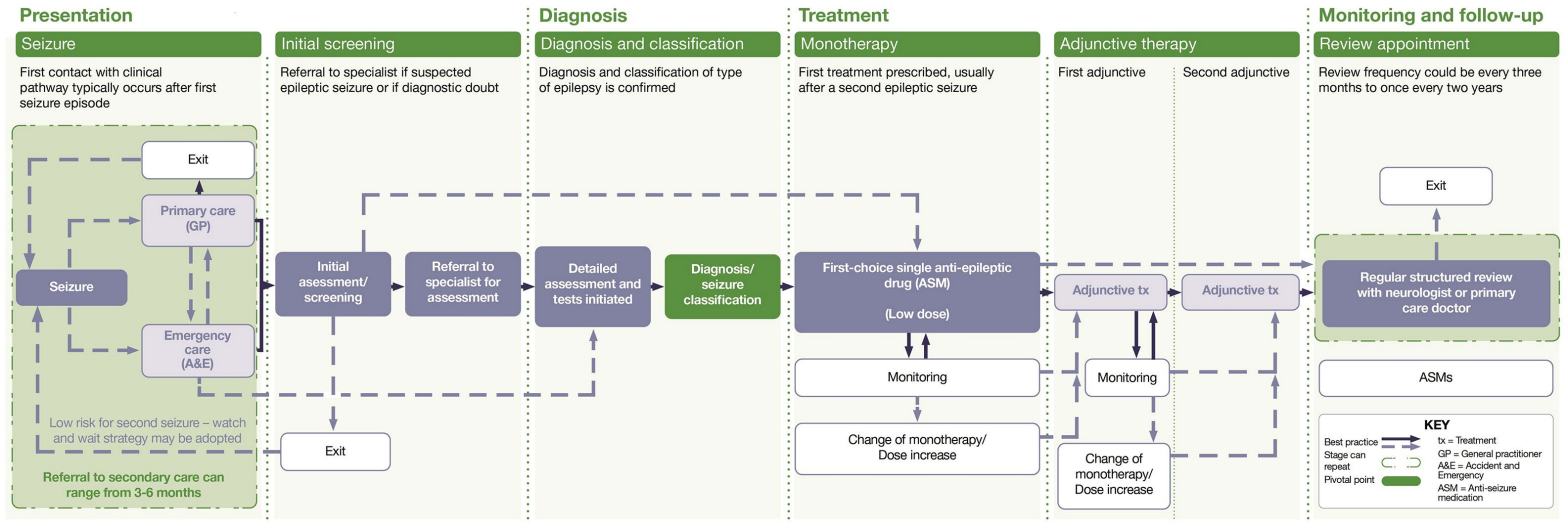
Clinical journey map.

Figure 1 displays the distinct clinical stages of the disease journey for people with epilepsy in Europe, from presentation through to monitoring and follow-up stages. The journey is not always a linear one; it can vary significantly between people with epilepsy, with some people not progressing through the different stages.

### 3.3. Emotional patient journey map

**Figure 2:**
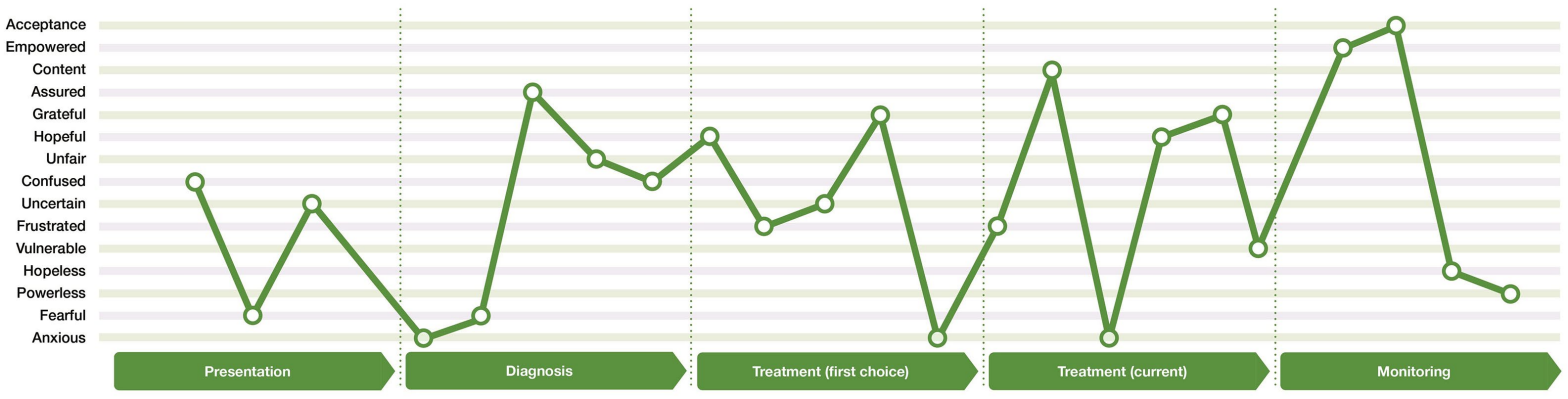
Emotional journey map.

Figure 2 illustrates an aggregated emotional journey map developed from the participants’ reported emotions at each stage of the clinical journey. The lines that join the different emotions, do so only to illustrate the extent of the highs and lows of emotions that can be experienced. This emotional journey map is an aggregate of the emotional experiences of participants, and does not mean that all people with epilepsy experienced all these emotions, nor does it mean that these are the only emotions that each participant expressed or experienced. Furthermore, it does not mean that the emotions were experienced in a set order from one emotion to the next. However, it does illustrate how the participants may experience the same or similar emotions at different stages of the journey, or experience both positive emotions (e.g., gratitude) and negative emotions (e.g., frustration) within the same stage of the journey (e.g., treatment [first choice]). The emotional journey map therefore illustrates the emotional rollercoaster that people with epilepsy can experience.

### 3.4. Thematic analysis within the journey stage

Data (e.g., patient quotes) from each of the 40 interviews were extracted and coded into the most relevant section within the clinical map framework (Table 3). Due to analyzing the patient experience per journey stage, similar themes may occur in more than one stage (e.g., feelings of uncertainty or the impact of the doctor-patient relationship).

**Table 3:** Themes and sub-themes within each of the four clinical stages of the epilepsy journey.

Epilepsy journey for people on polytherapy			
Stage 1: Presentation – Life is turned upside down	Stage 2: Diagnosis – Period of learning	Stage 3: Treatment – Aspirations and experimentation	Stage 4: Monitoring and follow-up – Feeling “out on a limb”
Symptom recognition	Positive encounters	Treatment initiation	Ongoing symptoms
Entering the system	Searching for the root cause	Treatment changes	Ongoing emotional and psychological support
	Involving the child	Adherence and side effects	Impact on QoL
	An uncertain future	Non-adherence	Stigma and discrimination
	Role of psychological support	Period of adjustment	
	Stigma and lack of awareness	Information provision	
		Emotional support	

QoL, quality of life.

#### 3.4.1. Stage 1: Presentation – Life is turned upside down

##### 3.4.1.1. High variability of symptoms contributes to a lack of symptom recognition

The unique symptomology of seizures meant that the way participants first presented varied from person to person. For many, their first seizure happened spontaneously, and occasionally, in a dangerous situation (e.g., in the bath). Others reported having symptoms before their first seizure, including fainting, headaches, sweating, jerks, and phantom odors and tastes.

There was a low awareness of the symptoms of epilepsy among many of the participants; for example, many had only heard of tonic–clonic seizures, and did not recognize the signs of other types of seizures. Seizures accompanied by loss of consciousness might have initially been attributed to fainting, stress, or low blood pressure, and were usually recognized as a symptom of epilepsy in hindsight, post diagnosis.

*“It started with a shaking and twitching of My leg. I could Not control It. First, I thought it was because of overstraining. I did not think of epilepsy...” [participant 4.1, Germany]*

##### 3.4.1.2. Challenges of entering the system cause confusion and fear

Participants were either taken to the emergency department following a first seizure or to their family practitioner/general practitioner, dependent on presentation; for example, people who experienced absences and loss of consciousness, usually saw their primary care provider, whereas those with other types of seizures went to the hospital. The participants that were taken into the emergency department were often left confused about their possible diagnosis and feared the potential outcome.

*“…very nervous, anxious, tense, and worried. I was an emotional wreck. I did not know what was happening, why I was hospitalized, what they were doing.” [Participant 3.3, Spain]*

Most participants who were diagnosed as children could not remember their life experiences pre diagnosis, but remembered feeling sad, scared, and being unsure of the reason for their tests/scans. Many were concerned about being different from their peers, and anticipated peer rejection. Participants who were children at this stage, reported that they had missed school and experienced disruption in their social life because of initial testing and treatment.

Participants who were adults when they had their initial investigations, experienced anxiety in anticipation of the outcome of their diagnosis; for example, concerns were often related to their belief that they were experiencing a stroke or psychosis, or had a tumor or cancer. Delays in diagnosis left them with feelings of uncertainty and a lack of information about their seizures and their management.

At this stage, participants reported significant disruption to their lives. They had feelings of being “out of control” and being debilitated by the lack of certainty around their daily lives.

*“…it came very suddenly; it was a drastic change, and shock, no longer able to drive, to continue ‘a normal life’.” [Participant 1.1, UK]*

Furthermore, participants recalled being confused while in the hospital and unable to remember what the healthcare professional had explained.

*“[I] didn’t understand what was happening to me, I felt like I was going crazy.” [Participant 2.3, Italy]*

#### 3.4.2. Stage 2: Diagnosis – Period of learning

##### 3.4.2.1. Positive encounters with healthcare professionals drive emotional well-being

Positive diagnostic experiences were characterized by positive encounters with neurologists/epileptologists, highlighting the importance of this relationship. Participants who spent more time with their neurologist/epileptologist felt they had the information needed to understand their epilepsy. Participants generally had frequent appointments at this stage, and relied on and trusted their neurologist/epileptologist. Those who felt reassured and supported by their core healthcare professional, were able to adapt and adjust to their diagnosis.

*“I did feel supported; I had a very quick appointment, didn't feel left on my own […] high confidence towards my neurologist.” [Participant 1.1, UK]*

*“She [my neurologist] explains things so that everyone can understand […], and she is also able to take away the fear and panic.” [Participant 4.4, Germany]*

Participants highlighted that meeting others with epilepsy at diagnosis would have benefitted them, but they did not always get the opportunity. Participants who were able to meet others with epilepsy while in hospital felt less alone.

“*I would have appreciated meeting fellow patients to learn and know what living with epilepsy is like.” [Participant 3.5, Spain]*

##### 3.4.2.2. Difficulties understanding the epilepsy diagnosis

Participants wanted to know the cause of their epilepsy. Healthcare professionals’ explanations of the diagnosis were often complex and hard to understand, with many wanting the neurologist/epileptologist to “come down to their level.” They felt that a dedicated healthcare professional who could speak to them on a personal level, consider social aspects of their life, and spend time to reassure and discuss information with, would have been beneficial. Not understanding the cause and diagnosis triggered frustration.

*“[…] but the main question is the real root cause of the epilepsy. I had it for 35 years and I would like to know the origins of it.” [Participant 5.5, France]*

##### 3.4.2.3. The importance of the caregiver

For participants diagnosed in childhood, parents and caregivers had been their main source of information. Involving the child in the diagnostic process by helping them in understanding their illness, was recommended as a strategy to reduce their confusion.

*“It would be good to involve children directly or as early as possible, as their parents might not do it, as in my case.” [Participant 4.7, Germany]*

##### 3.4.2.4. Uncertainty about the future causes frustration and worry

There was little opportunity to explore what the diagnosis meant for the participants’ day-to-day life. Participants had questions about repeated seizures and the possibility of brain damage. Many felt that the discussions with healthcare professionals and information provided to them focused on what the person should avoid, instead of what they were still able to do, and some of the terminology used could be perceived as scary. Participants were unsure about their future ability to drive, have children, drink alcohol, and continue to work. This left them feeling overwhelmed, shocked, and with a sense of injustice, from epilepsy stripping them of future opportunities.

*“It was all very overwhelming because it was a list of things I could not do, rather than reassuring.” [Participant 3.8, Spain]*

*“I was shocked when I was told that I could not continue my apprenticeship as a carpenter, as it involves the handling of machines.” [Participant 4.5, Germany]*

*“…before the diagnosis, I had a life full of sporting initiatives, of dreams to realize; all this collapsed after the diagnosis.” [Participant 2.7, Italy]*

*“I was told that I had a ‘mini storm’ in my mind, which I find extremely scary.” [Participant 5.2, France]*

##### 3.4.2.5. Role of psychological support

Experiencing mental health issues was common, as many of the participants expressed difficulty adjusting to their diagnosis, including panic attacks, anxiety, and depression. Those who were offered psychological tools and support by their healthcare professionals found it helpful, but others had to look for support from psychologists outside the hospital. Some deemed professional support unnecessary for them personally, however, retrospectively felt that they would have benefitted from support. Outside the healthcare setting, a strong family network could help people cope with mental health issues and their diagnosis.

*“I had panic attacks, anxiety, depression, and all that was totally overlooked.” [Participant 3.5, Spain]*

*“It would have been nice to have it. Now looking back, I realize that they [healthcare professionals] take for granted that you are mature enough to digest everything they tell you. And I was not.” [Participant 3.8, Spain]*

##### 3.4.2.6. Stigma and lack of awareness

Stigma was reported to come from multiple sources, including healthcare professionals, friends, family, peers, and the public. Participants reported feeling fearful or embarrassed by public perceptions of epilepsy, and felt that there was a lack of awareness of the condition and its symptoms.

*“Someone affected by epilepsy doesn’t only suffer from the condition itself, but also from the lack of education of everybody else around them.” [Participant 2.1, Italy]*

#### 3.4.3. Stage 3: Treatment – Aspirations and experimentation

##### 3.4.3.1. Treatment initiation Is associated with hope and uncertainty

Participants were generally initiated onto a single, low-dose ASM (monotherapy) following diagnosis. Treatment aims ranged from reducing the number and/or intensity of seizures to eliminating them entirely, with little to no medication side effects. Participants’ expectations were therefore often optimistic, and they were hopeful they would be able to resume life as before, or at least see an improvement in their QoL. This sense of hope and optimism was often accompanied by a sense of uncertainty about the future, as participants were aware there was no guarantee that their first-line monotherapy treatment would work or continue to work for them in the long term. Those with a good treatment response, on their current treatment, were able to return to their daily routine, and expressed gratitude that they had experienced respite from seizures.

*“I hoped that this would have been the right one…” [Participant 1.6, UK]*

*“I hoped [with treatment] to have a normal life, be able to work again. Not be dependent on welfare.” [Participant 4.8, Germany]*

*“[…] they were very vague, saying that I could have a seizure tomorrow, next week, next year or never.” [Participant 3.3, Spain]*

“I have not had a seizure in years… No longer on the verge of the cliff.” [Participant 3.1, Spain]

##### 3.4.3.2. Treatment changes can cause confusion

Whether the participant remained on the prescribed treatment regimen, depended on the outcome of the regular review (clinical monitoring and an assessment of the impact of epilepsy and medication on the person’s daily life). If the frequency/intensity of seizures did not improve, there was a drop in effectiveness over time (e.g., months or years), or the person did not tolerate the treatment, then a trial-and-error approach was taken. This process, decided by the participant’s assigned neurologist/epileptologist, involved a gradual increase of the current ASM dose, a new single ASM, or an add-on therapy.

Treatment changes could cause confusion, however, when established on a new treatment regimen, participants felt hopeful that their treatment would be successful in stopping seizures and improving their QoL. They initially felt content in their new therapy, and expressed feeling safe, supported, and confident that they would be seizure free.

While some accepted that they might need to start and stop multiple treatments to reach and maintain control of their epilepsy, others were left with feelings of frustration, disappointment, and hopelessness regarding the possibility of continued treatment failures.

*“… it was a bit done by trial and error.” [Participant 5.2, France]*

*“[I expected] a normal life [from the new treatment regimen]. I expected a better quality of life and not all the side effects.” [Participant 2.1, Italy]*

*“When I got the present combination, I felt it was a miracle, felt so happy, everything had gone back to normal […] very grateful for it, no negative impact.” [Participant 1.1, UK]*

*“Now I am so well adjusted that I hardly ever have a seizure, maybe once a year or twice at the most. I am content with my therapy, and I don’t want to switch to another drug.” [Participant 4.7, Germany]*

*“Every time they [added] a pill, it felt like they were improving the treatment, that I was being better taken care of, and that maybe, one day this will all end.” [Participant 3.3, Spain]*

*“It was a quite different treatment regimen. [If it were up to me, I would] just want to carry on with my life, free of all the treatments. It was [disappointing] to an extent, because I feel less […] hopeful regarding the treatment options.” [Participant 1.5, UK]*

*“…I felt unstable. I felt sad and angry… I didn’t feel optimistic because it was very difficult and challenging to move from one drug onto another. That’s because your whole body is [on] this treatment, and it can cause head-spinning episodes, eyesight problems, [anxiety], insomnia … Your whole body is all over the place.” [Participant 2.8, Italy]*

##### 3.4.3.3. Side effects impact on QoL

Participants reported experiencing a range of side effects. Those who perceived their medication as the key to controlling their seizures, tended to accept medication side effects as a “price worth paying,” yet others found the side effects hard to live with. Experiencing side effects could prompt the participant to discuss medication changes with their healthcare professional. However, healthcare professionals were not always sympathetic, in particular concerning side effects that were perceived to be less debilitating. Many who experienced medication side effects felt frustrated with the impact they had on their QoL, as well as worried about potential short-term negative consequences.

*“It is difficult emotionally, as well as physically, with side effects and issues at the memory level, and the impact on work.” [Participant 1.6, UK]*

*“I was falling asleep everywhere. I felt I could be robbed.” [Participant 3.2, Spain]*

##### 3.4.3.4. Non-adherence caused by concerns and doubts about necessity for treatment

As a consequence of side effects perceived to be intolerable, some skipped doses, experimented with their dosage, or stopped taking their medication altogether to experience temporary relief without consulting their healthcare professional. This non-adherence resulted in hospitalizations for some participants.

Concerns about potential side effects of ASMs were frequent among participants, and with some potential side effects resembling symptoms of the illness itself (e.g., memory loss or fatigue), some participants may have misattributed epilepsy symptoms as side effects (“nocebo effect”). To avoid misattribution, one participant commented that they would not read the patient information leaflet in the medication box.

*“The biggest challenge is exhaustion; it can put me in a bad mood, and sometimes I may skip one treatment. Sometimes I need a breather.” [Participant 1.5, UK]*

*“I stopped treatment for three days to see what the effects would be.” [Participant 5.1, France]*

*“At the beginning, it was really hard, and taking the treatment was an ordeal […], but I found it really hard to the point that I often just pretended not to have [epilepsy]… At some point, I stopped taking all drugs, until one day, I collapsed and ended up in hospital.” [Participant 2.3, Italy]*

*“Only months after receiving a new drug treatment, I would read the information leaflet. I did not want to read it prior, as I wanted to be open and not prejudiced.” [Participant 4.6, Germany]*

##### 3.4.3.5. Period of adjustment to medication changes

Participants often went through an expected period of adjustment when initiating a new medication regimen. Those that had a good relationship with their healthcare professional, were more likely to have trust or faith in the new medicine. However, if a person experienced a side effect while on a new medication regimen, it could lead to them feeling defeated, despite the quality of the relationship with their healthcare professional.

Add-on therapies could lead to participants experiencing practical medication challenges in their day-to-day lives, such as coping with the number of pills, remembering to take their medication, dealing with physical attributes of the medication (e.g., pill size), and having limited access to certain medications. However, participants often adjusted, and found strategies to help them cope.

*“Going from one drug to two was not an issue. I have always trusted her and her decisions. If she said it was the right thing to do…” [Participant 3.1, Spain]*

*“Every new seizure brings you down…” [Participant 1.1, UK]*

*“I bought myself a weekly pill box because a couple of times I couldn’t remember whether I’d already taken the pill or not, and this can cause problems… So, I got organized with the weekly pill box.” [Participant 2.2, Italy]*

##### 3.4.3.6. Lack of information and conversations about treatment options

While some participants felt satisfied with the depth and quality of information and support they received from their healthcare professionals, others reported the opposite. They often did not receive written information about the current treatment to manage their expectations (i.e., risks and benefits), but instead, were provided with verbal information that they would later forget. Participants reported limited discussion with their neurologist/epileptologist about treatment changes. Although some felt that their opinion was taken into consideration, others did not feel part of the decision-making process, or decided to leave decisions entirely to the neurologist, as they did not feel equipped to contribute.

Participants reported looking for further information *via* the internet, including patient advisory group websites, patient forums, peer-reviewed journal articles, and drug company websites, to find the information about a specific treatment change. Participants were particularly interested in information about medication side effects and possible treatments for counteracting side effects.

*“Very little [information] besides some [verbal] information during phone calls on potential side effects. In general, there is a lack of upfront information.” [Participant 1.2, UK]*

*“I was not offered options. Her words were ‘I am going to try this’ […] I have always been the kind that blindly follows what the doctor says. I sometimes do not even ask a single question.” [Participant 3.7, Spain]*

##### 3.4.3.7. Emotional support

Family members and close friends could be a helpful source of emotional and mental support while participants adjusted to a new medication regimen. For some, the idea of accepting support from outside the family was unthinkable, but most felt that it would have been beneficial to have additional support available from the healthcare system/hospital. Psychological support was rarely offered as part of a treatment package, sometimes resulting in the person feeling abandoned. When it was offered, it was often viewed as inadequate, due to long waiting lists, or it only being offered for a short period without continued support. Psychological support usually involved talking to a healthcare professional, either face to face or *via* the telephone, about their medication changes, to better manage their expectations and fears between consultations.

*“They ask you if you need emotional support. You know, though, that there are no appointments with psychologists until one year later, unless you have something really urgent.” [Participant 4.8, Germany]*

*“… [I would have liked] having someone to talk to, even just a nurse to process the changes, the expectations, and having a bit [of] conversation.” [Participant 1.3, UK]*

#### 3.4.4. Stage 4: Monitoring and follow-up – Feeling “Out On a limb”

##### 3.4.4.1. Ongoing symptoms and support

Fully establishing a satisfactory treatment regimen could take time. Therefore, despite receiving treatment, participants often still reported symptoms of epilepsy and side effects from their medication.

Although some participants felt that they continued to receive good support from their healthcare professionals, others felt neglected and abandoned at this stage due to fewer touchpoints with their specialist team. Those participants reported that their support network was made up mostly of close family and friends, and while some were satisfied with that level of support, others lacked a social support network.

*“No support whatsoever. Epilepsy is not regarded as a long-term systemic disease, rather something [patients] ought to manage on their own.” [Participant 1.6, UK]*

##### 3.4.4.2. Impact of epilepsy on QoL

Participants reported that their epilepsy had a negative impact on all aspects of their QoL and psychological well-being at some point in their patient journey. All key areas of a participant’s life could be affected long term, including, but not limited to, socializing, sleep quality, working, using transport, and traveling. These functional limitations could leave the person with epilepsy feeling that they had lost their freedom, and depended on others for help and support. Those on a successful treatment regimen, however, reported learning to adapt and beginning to rebuild their lives.

*“I would describe it like having a heavy stone on my back. Something heavy and attached to my back that I can’t get rid of, and the more I try to discard it, the more it hangs on to me.” [Participant 2.8, Italy]*

*“It’s been a huge journey and lots of work has been done. With Depakin®, I became more socially engaged, I studied more, I read more… I went out more often, and I learned to relate to other people again…” [Participant 2.1, Italy]*

Personal relationships could be especially challenging, and participants’ social life could feel restricted in several ways. For example, participants reported avoiding to socialize, due to fearing they might get overtired or become exposed to flashing/strobe lights, which could trigger a seizure. Furthermore, they reported that friends did not always understand their condition, and they could no longer relate to their old friends, now that their lives had changed so much. An inability to be spontaneous was another reported barrier to socializing.

*“I don’t feel confident enough to [be spontaneous]. For example, if a friend says […]: ‘Do you feel like going out for dinner tonight?’ I think: ‘Oh no, you did not have a nap, did not sleep for [enough hours], and what if you feel tired all of sudden and get a seizure?’. I wouldn't want to put anyone through that.” [Participant 4.8, Germany]*

Sleep disorders were frequently reported (e.g., insomnia, sleep apnea, and restless leg syndrome), and lack of sleep could be a trigger for a seizure.

*“I got to a point [where] I have to accept epilepsy as part of who I am. Tiredness is horrific, [particularly] with children. I try to sleep in the afternoon – a nap to be able to cope with epilepsy and avoid blackouts, or even seizures.” [Participant 1.5, UK]*

Using transport and traveling could also be impacted. Participants who still experienced seizures, reported that they were not able to drive, and traveling far from home was complex and required detailed planning. Participants reported that they consequently often chose to stay closer to home than they used to prior to their epilepsy diagnosis.

Participants also reported that they had to stop working because of continued symptoms, or due to side effects of their ASMs (unemployment). Others reported only working part time on jobs below their skillset and capabilities (underemployment), or having to find alternative employment, especially if their previous work involved an element of driving or handling large machinery. For many, their job was important for their sense of self-worth and their limitations due to their epilepsy impacted their mental health and sense of self. Other participants did not feel that their jobs or careers had been hampered due to their epilepsy; however, they still experienced constant worry about having a seizure at their workplace.

*“I am not able to drive; I have absences, lapses – it is nearly impossible for me to have a job, not only from the epilepsy, but [also] from the side effects of treatment.” [Participant 5.6, France]*

##### 3.4.4.3. Stigma and discrimination

As reported in earlier stages, participants could experience continued long-term stigma and discrimination in their everyday life, as there was little reported awareness of the illness among the public. Participants coped with the stigma by either talking about their condition openly, or not discussing it at all when they did not have to. Participants also reported experiencing discrimination in the workplace or in public places.

*“It looks like you are drunk, and people would leave you in the middle of the street; they are not told about [this condition]. There should be more awareness campaigns.” [Participant 1.5, UK]*

## 4. Discussion

Content and framework analysis of semi-structured interviews with 40 people with epilepsy on polytherapy, has identified their psychological and emotional journey (Table 3), key insights along the disease journey (Table 4) and unmet needs (Table 5).

**Table 4:** Key insights at each stage of the patient journey.

Stage 1: Presentation – Life is turned upside down	Stage 2: Diagnosis – Period of learning	Stage 3: Treatment – Aspirations and experimentation	Stage 4: Monitoring and follow-up – Feeling “out on a limb”
The pathway to diagnosis differed among patients. The unique symptomology of seizures meant the way they first presented varied from person to person	The quality of the relationship between the healthcare professional and patient, impacted the patients’ emotional experience, understanding, and ability to self-manage	Treatment decisions were often not collaborative. Patients reported limited discussion about treatment	Patients could feel “neglected” or “abandoned” by the healthcare system once in the monitoring stage
Hospitalization was a traumatic experience for many of these patients	Understanding the cause of their epilepsy was important to patients, and they wanted to be provided with a tailored explanation	If first-line monotherapy did not work, then a trial-and-error approach was taken to find the best medication, combination of medications, and/or dose	Patients experienced living with epilepsy in very different ways. Some adjusted, whereas others did not, and it continued to impact QoL
–	Parents and caregivers played a crucial role for children diagnosed with epilepsy	Some patients stopped taking or reduced their medications at some stage of their treatment without consultation with their healthcare professional	Epilepsy can be a hidden disability, and therefore misunderstood. Patients reported that there was low social awareness

QoL, quality of life.

**Table 5:** Key unmet needs at different stages of the epilepsy journey.

Presentation and diagnosis	Treatment	Monitoring and follow-up
Lack of adequate information, support, and resources for patients for next clinical steps, as well as for seizure and other epilepsy symptom recognition	Lack of shared decision-making between the patient and the neurologist about treatment. Patients reported that they did not always receive adequate information on the risks of each treatment, and why a treatment had been selected	Patients felt that they were left with inadequate support, and that they needed to seek out and arrange their own support
Lack of communication between patients and healthcare professionals during diagnosis, and a missed opportunity for further exploration of diagnosis	Patients were making unplanned changes to the medication dose or frequency without first consulting their prescribing healthcare professional. Non-adherence is likely to have a negative impact on treatment outcomes	Patients might still be left with many unanswered questions, leaving them feeling unsettled and struggling to accept their illness
Lack of information and resources post-diagnosis. Patients relying on external sources post-diagnosis, causing uncertainty about the impact on daily life and health outcomes	Lack of clarity on how epilepsy specialists decided which monotherapy or add-on therapy/therapies to prescribe	Much of the emotional and psychological support a patient received was provided by their close family members, rather than from healthcare services

### 4.1. Implications for key stakeholders and the healthcare system

Thematic synthesis, within the four clinical stages, resulted in emergent themes that have helped to identify potential targets for future quality improvement interventions. Unmet needs were identified at multiple levels, including at the individual level (e.g., medication non-adherence); the healthcare professional level (e.g., lack of education provision about seizure care to the person with epilepsy); the healthcare system level (e.g., lack of prescribing guidelines for when first-line monotherapy therapy does not work); and the wider community level (e.g., low public awareness/lack of public awareness campaigns).

There are five overarching recommendations for future consideration, based on the unmet needs identified from the current study’s findings. It is important to note, that we do not claim that these are unique recommendations, and some or all, might already be under consideration or development within clinical practice or through patient organizations.

#### 4.1.1. Establish and promote support networks from presentation through to monitoring and follow-up stages

This study highlighted the psychosocial burden of living with epilepsy. There is a need for consistent psychological support for people with epilepsy and caregivers at all stages of the patient journey, from presentation through to monitoring and follow-up stages. The call for support networks to be established in the epilepsy care pathway is echoed by other research (19, 33). There is also an opportunity to develop and evaluate psychosocial interventions that support people who are experiencing difficult emotions that relate to living with epilepsy, in the same way that previous research has recommended in the case of sub-populations, such as the elderly (17) and those with a previous psychiatric history (16). Furthermore, in a paper by Kemp et al. (5), they argued that there is an opportunity not only to support patients with their distress, but also to encourage the use of positive psychology and cognitive behavioral therapy to promote mental health well-being in people living with chronic conditions (5).

#### 4.1.2. Accelerate pathway to diagnosis

The findings from this study suggest that, at least from the patients’ perspective, there is a lack of clarity about what constitutes appropriate seizure care within the healthcare system, and this appears to have the potential to delay the pathway to diagnosis and treatment. Although there have been recent developments in the diagnosis of epilepsy using technological advancement (34), it would still be advantageous to provide patients entering the healthcare system with standardized education on: how to recognize seizures and other symptoms of epilepsy; seizure first aid; how to report any further seizures; and details of next steps. Furthermore, although not identified as an unmet need in the current study, Watson et al. (19), identified that there were practical barriers to access testing facilities, and they recommended the provision of at-home testing, dedicated travel assistance programs, and enhanced education on the importance of prompt testing, which would help to prevent treatment delays (19).

#### 4.1.3. Provide opportunities to discuss the diagnosis with patients

When a person is diagnosed with epilepsy, it is essential that they are able to discuss their diagnosis with their neurologist/epileptologist. In our study, we found that participants were more likely to have a positive diagnostic experience, a better understanding of their condition, and a better ability to self-manage, if they felt that they had this opportunity. Fazekas et al. (18), argued that there may even be a lack of awareness or sensitivity among healthcare professionals, who may consequently underestimate the concerns of people with epilepsy, or not take sufficient time to discuss their needs (18). It is crucial that people are given ample time and space to discuss their diagnosis with their neurologist/epileptologist or other relevant healthcare professionals.

#### 4.1.4. Clarify treatment change guidelines for patients

Although the participants in this study were on polytherapy at the time of their interviews, their experiences during presentation and diagnosis are likely to be similar to a person for whom first-line monotherapy treatment was successful. Differences in experiences would likely only occur during the treatment stage of the clinical journey, because of the additional burden of the trial-and-error approach to medication that patients perceive to take place in clinical practice. The analysis suggests that there is a lack of clarity, for patients, on how epilepsy specialists have reached a consensus on what monotherapies or add-on therapies to prescribe. Therefore, it is important to include the patient in the treatment choice process through shared decision-making and general engagement, such that the healthcare professional and their patient are involved in the co-creation of a care package. Further research to ascertain the impact of implementing shared decision-making and patient engagement tools in establishing the patient journey in epilepsy is warranted.

#### 4.1.5. Development of a patient engagement and shared treatment decision-making tool

Treatment decisions are not always collaborative, and people with epilepsy can stop taking or reduce their medication without consulting with their healthcare professional. This might be due to misperceptions they may hold about their treatment (intentional), or as a consequence of practical/physical barriers to taking their prescribed medication (unintentional). Either way, it would be beneficial if healthcare professionals adapt their consultation style to the needs of the individual to address non-adherence in a non-judgmental way (35). We recommend raising shared decision-making and patient engagement awareness among healthcare professionals, and propose the development of a toolkit for healthcare professionals and people with epilepsy to prompt discussions about their treatment and epilepsy self-management, including add-on therapies, non-pharmacological treatments, and adherence. Empowering people with epilepsy to speak up, and not just passively receive their treatment regimens, is likely to improve patient–provider relationships, as well as their health outcomes, especially since non-adherence could be contributing to the deterioration in seizure control (36).

Within this decision-making and engagement tool, it would be imperative to educate patients about treatment options, processes, and managing expectations of ASMs. The treatment goal of an ASM is to achieve complete seizure freedom, or at least to reduce the frequency and/or intensity of seizures. In order to set the right expectations, it is essential to inform patients that there are other symptoms associated with epilepsy (comorbidities) that might require additional treatments, other than the ASM.

### 4.2. Limitations and recommendation for futures research

Limitations to this study should be acknowledged. First, there are limitations within the sample. Considering this was a qualitative interview study, we did access a relatively large sample of people living with epilepsy on polytherapy (*N* = 40) in five European countries, yet due to the country spread, it was limited to eight participants per country, each with a mix of age of onset and seizure types. Although there is no specific reason to believe that the subjective experiences expressed by the current sample would differ significantly from other people’s experience within the same countries, it is possible there are differences across people with different clinical characteristics. For example, it is noteworthy that the greatest proportion of patients in this study reported having tonic–clonic seizures, and their experiences may differ to those who experience other types of seizures, such as absence seizures. Furthermore, it is possible that patients who had a psychiatric condition prior to diagnosis have more difficulties adjusting to their diagnosis compared with those that did not. This was touched on in a previous study that found that a higher proportion of patients with a previous psychiatric history described a pervasive loss of control, following diagnosis (16). However, we did not believe that the current study comprised sufficient individuals within any one category to merit our breaking down the analysis to identify any potential differences across participants. Second, qualitative analysis depends on researchers’ interpretations of textual data, and is therefore potentially open to bias and subjectivity; regular meetings between researchers to discuss and verify the ongoing coding, aimed to limit any confirmation bias. In addition, illustrative quotes have been presented throughout to demonstrate the validity of our analysis. Third, not all participants in this study experienced the same intensity of emotions throughout their journey, nor did all the participants experience the same emotions at the same stage. Not taking these differences into account would be misleading. The analysis offers only an overview of the potential emotional, psychological, and social experiences, and identifying disparities in such experiences across participants, was not an objective of this study. Patient demographics and clinical presentations varied, such as time since diagnosis and type of ASM. Future research could endeavor to investigate whether any differences in emotional, psychological, and social experiences identified in the current study, could reliability be associated with socio-demographic characteristics, such as gender, comorbidities, country of residence, type of epilepsy, or degree of social support.

### 4.3. Conclusion

Our research provides a comprehensive overview of the experiential journey that people with epilepsy on polytherapy can experience. The emotional mapping exercise specifically illustrates the emotional rollercoaster that many people with epilepsy experience. Our analysis has identified themes in each stage of the clinical journey, from presentation through to monitoring and follow-up stages, and provided actionable recommendations ranging from an individual level to a healthcare system level. Findings have the potential to drive change at the healthcare level, and stop the emotional rollercoaster from derailing.

## Data availability statement

The datasets presented in this article are not readily available because of ethical and privacy restrictions. Requests to access the datasets should be directed to the corresponding author.

## Ethics statement

Ethical review and approval was not required for the study on human participants in accordance with the local legislation and institutional requirements. The patients/participants provided their written informed consent to participate in this study.

## Author contributions

EG-R conducted the analysis and interpretation of the interview transcripts, as well as wrote the manuscript. CK contributed to the conceptualization of the study design, the management of the analysis and interpretation, as well as provided input into revising and editing of the manuscript. SR contributed to the conceptualization of the study design, as well as provided input into the interpretation of the data, and into editing of the manuscript. AA led the acquisition of patients and patient interviews. She also contributed to the conceptualization of the study design. LB contributed to the conceptualization of the study design, as well as provided input into revising and editing of the manuscript. RS-F contributed to the conceptualization of the study design, as well as provided input into revising and editing of the manuscript. BS was the principal investigator of the project. She contributed to the conceptualization of the study design, as well as provided senior input into all aspects of the study from conception to completion. All authors contributed to the article and approved the submitted version.

## Funding

This study was supported by Eisai Europe Ltd.

## Conflict of interest

LB, RS-F, and BS were employed by Eisai Europe Ltd.

The remaining authors declare that the research was conducted in the absence of any commercial or financial relationships that could be construed as a potential conflict of interest.

## Publisher’s note

All claims expressed in this article are solely those of the authors and do not necessarily represent those of their affiliated organizations, or those of the publisher, the editors and the reviewers. Any product that may be evaluated in this article, or claim that may be made by its manufacturer, is not guaranteed or endorsed by the publisher.
